# Ochre star mortality during the 2014 wasting disease epizootic: role of population size structure and temperature

**DOI:** 10.1098/rstb.2015.0212

**Published:** 2016-03-05

**Authors:** Morgan E. Eisenlord, Maya L. Groner, Reyn M. Yoshioka, Joel Elliott, Jeffrey Maynard, Steven Fradkin, Margaret Turner, Katie Pyne, Natalie Rivlin, Ruben van Hooidonk, C. Drew Harvell

**Affiliations:** 1 Department of Ecology and Evolutionary Biology, Cornell University, Ithaca, NY 14850, USA; 2 Department of Health Management, University of Prince Edward Island, Atlantic Veterinary College, Charlottetown, Prince Edward Island, Canada C1A 4P3; 3 Department of Biology, University of Puget Sound, Tacoma, WA 98416, USA; 4 Laboratoire d'Excellence «CORAIL» USR 3278 CNRS-EPHE, CRIOBE, Papetoai, Moorea, Polynésie Française; 5 Lake Crescent Laboratory, Olympic National Park, Port Angeles, WA 98362, USA; 6 Marine Science Center, Northeastern University, Nahant, MA 01908, USA; 7 Atlantic Oceanographic and Meteorological Laboratory, NOAA, 4301 Rickenbacker Causeway, Miami, FL 33149, USA; 8 Cooperative Institute for Marine and Atmospheric Studies, Rosenstiel School of Marine and Atmospheric Science, University of Miami, 4600 Rickenbacker Causeway, Miami, FL 33149, USA

**Keywords:** epizootic, sea star wasting disease, *Pisaster ochraceus*, host demography, mass mortality, climate change

## Abstract

Over 20 species of asteroids were devastated by a sea star wasting disease (SSWD) epizootic, linked to a densovirus, from Mexico to Alaska in 2013 and 2014. For *Pisaster ochraceus* from the San Juan Islands, South Puget Sound and Washington outer coast, time-series monitoring showed rapid disease spread, high mortality rates in 2014, and continuing levels of wasting in the survivors in 2015. Peak prevalence of disease at 16 sites ranged to 100%, with an overall mean of 61%. Analysis of longitudinal data showed disease risk was correlated with both size and temperature and resulted in shifts in population size structure; adult populations fell to one quarter of pre-outbreak abundances. In laboratory experiments, time between development of disease signs and death was influenced by temperature in adults but not juveniles and adult mortality was 18% higher in the 19°C treatment compared to the lower temperature treatments. While larger ochre stars developed disease signs sooner than juveniles, diseased juveniles died more quickly than diseased adults. Unusual 2–3°C warm temperature anomalies were coincident with the summer 2014 mortalities. We suggest these warm waters could have increased the disease progression and mortality rates of SSWD in Washington State.

## 1. Introduction

Infectious disease outbreaks are a recurrent force structuring populations of many species. Most disease outbreaks are limited to a single host species or a related group of hosts [1,2]. In such cases, a host–pathogen evolutionary ‘arms-race’ often prevents pathogens from becoming so virulent that they cause host mortality, which can, in turn, prevent severe population declines or extinction [2,3]. In contrast, evolution of virulence in multi-host pathogens is less constrained and can cause rapid population declines in non-reservoir species. For example, chytridiomycosis, which occurs in over 350 species of amphibians, is associated with widespread population declines and extinctions around the globe [4]. Similarly, white spot syndrome, a virus of shrimp, affects over 78 decapod crustacean species and is the largest recorded pandemic in the ocean [5,6]. Recent global increases in multi-host high-impact diseases highlight the need to improve our understanding of the host and environmental factors that drive patterns of infectious disease [7,8].

In many wildlife diseases, it is challenging to unravel host, pathogen and environmental factors that influence the disease. One approach uses patterns in nature of infection or disease across host size or age [9], revealing information about how these players and their environment interact [10–12]. Differential impacts of disease on size classes can affect population recovery, especially in long-lived species where losses of reproductive adults can dramatically reduce the population growth rate for multiple generations [13]. These host–pathogen interactions are embedded in a changing environment, and environmental risk factors such as warming temperatures can alter these interactions [14,15]. In this paper, we report on dynamics of the ongoing sea star wasting disease (SSWD) epidemic, which began in 2013 and extends from Alaska to Mexico, impacting more than 20 species of asteroids. Previous asteroid wasting disease outbreaks were linked with temperature [16–18]. Strongly positive temperature anomalies developed in the northeast Pacific during the 2013–2014 northern winter and have persisted under the 2015 El Niño conditions. The anomalies documented were the greatest in the area since at least the 1980s [19,20], and it is unclear if warm temperatures may have exacerbated this outbreak.

**Figure 1.**
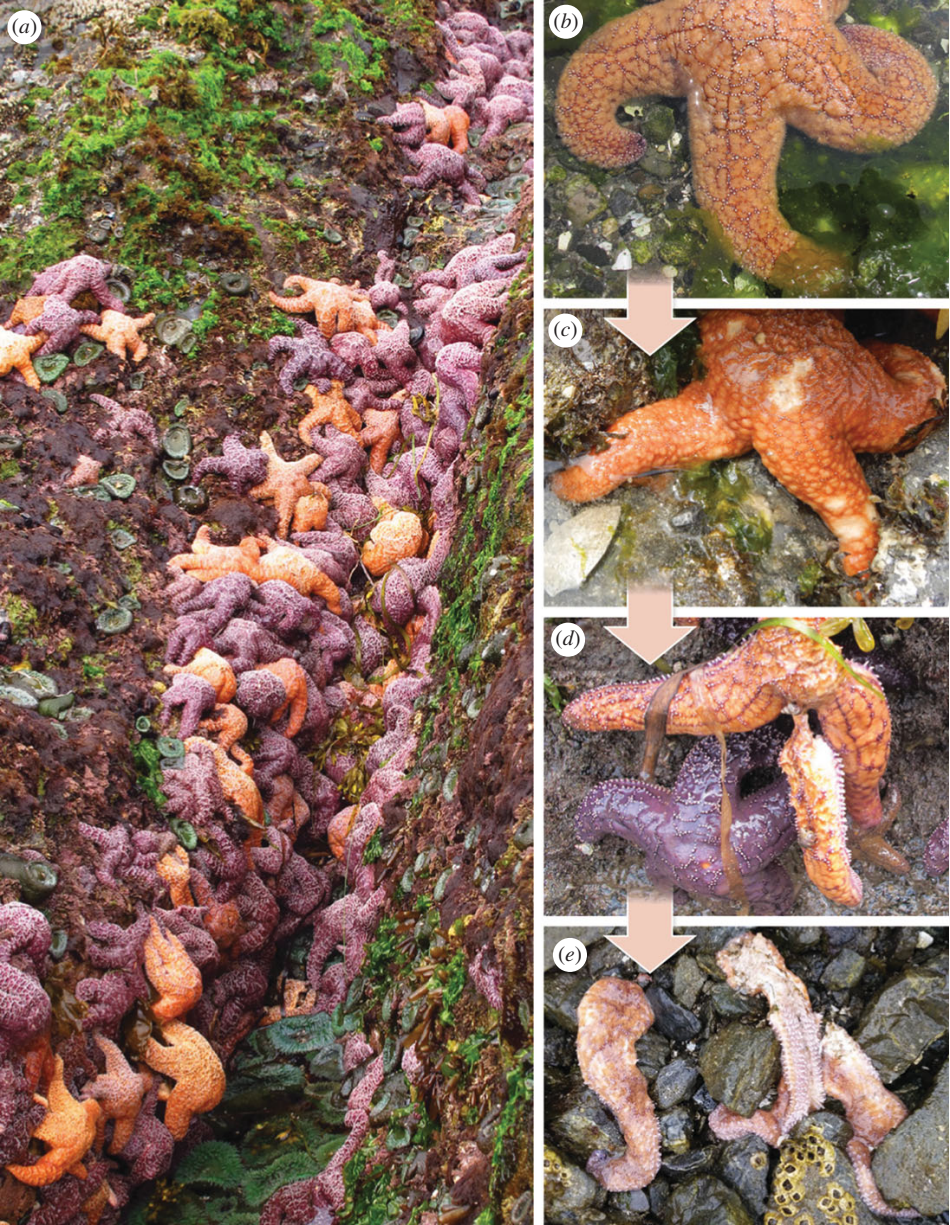
Representative photographs of *Pisaster ochraceus* pre-summer 2014 abundance and wasting disease progression. *P. ochraceus* were abundant in the intertidal survey areas in May, 2014 (*a*). Healthy sea stars (*b*) develop lesions (*c*) that can lead to arms detaching from central disk (*d*) prior to extensive tissue necrosis and death (*e*).

The current outbreak of SSWD resulted in a massive mortality event and is the largest documented marine disease epizootic of a non-commercial species to the best of our knowledge [21,22]. Sea stars with wasting disease develop lesions in the dermis that increase in depth and diameter, dissolving tissue from the outside in [16,22]. One or all arms then detach from the central disc as the sea star dies (figure 1*a–e*), often leaving only white piles of ossicles and disconnected limbs. Mass sea star mortalities during the 2013–2014 SSWD outbreak in California and Washington were linked to a sea star-associated densovirus (SSaDV, family Parvoviridae) using experimental challenge studies and a metagenomic analysis of field samples [22]. Previous outbreaks of SSWD have been reported to impact populations of focal sea stars species since the 1970s in the northeast Pacific, California and northern New England [16,18,23]. These earlier SSWD outbreaks involved a single species in a small region and were linked to departures in normal sea surface temperature [16–18]. In addition to at least 10 other species, the dominant stars affected in Washington State in this outbreak include: leather star (*Dermasterias imbricata*)*,* mottled star (*Evasterias troschelii*)*,* blood star (*Henricia* spp.), six-armed star (*Leptasterias hexactis*)*,* ochre star (*Pisaster ochraceus*), sunflower star (*Pycnopodia helianthoides*)*,* sun star (*Solaster dawsoni*)*,* slime star (*Pteraster tesselatus*)*,* rose star (*Orthasterias koehleri*) and Stimpson's star (*Solaster stimpsoni*)*.* The impact of the 2013–2015 outbreak on ochre sea stars is the focus for this paper because of the quality and consistency of the quantitative time-series data collected as well as its recognized ecological importance in intertidal rocky shores. The keystone species concept was founded on experimental demonstrations that predation by ochre sea stars structures rocky intertidal habitats in Washington [24,25].

The objective of this study is to characterize the outbreak timing, demographic impact and role of temperature in the SSWD epizootic in the northeast Pacific in 2014–2015. We use field surveys and experimental challenges to understand how SSWD progressed in three geographically distinct regions in and just outside of the Salish Sea in Washington (WA) state, USA. Specifically, we test the hypotheses that (i) disease prevalence and mortality are greater in larger size classes of sea stars, (ii) disease signs and mortality rate increases with temperature and (iii) breeding populations of sea stars declined after the SSWD outbreak.

## 2. Methods

### (a) Field surveys

**Figure 2.**
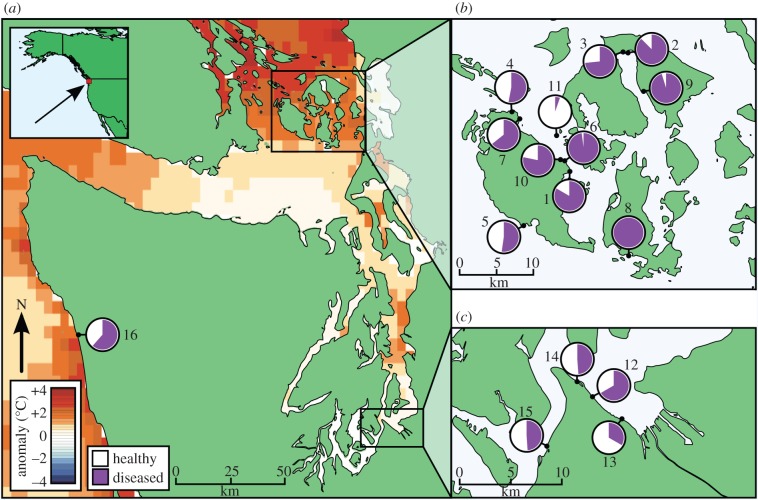
Site map shows peak prevalence at each region and 2014 summer sea temperature anomalies. A total of 16 sites were surveyed at (*a*) the Washington outer coast, (*b*) the San Juan Islands and (*c*) South Puget Sound of Washington (see electronic supplementary material, table S1 for coordinates). SST anomalies shown for the region are 5-km resolution and are average anomalies for June–August, 2014, with anomalies during each month calculated by comparing daily data to the monthly mean (calculated from 1985 to 2012). Pie graphs show maximum recorded adult SSWD prevalence over our study period. The sites with samples sizes (*n*) and the date when peak prevalence occurred are (1) Colin's Cove (*n* = 12, 7 August 2014), (2) Crescent Beach (*n* = 8, 9 August 2014), (3) Eastsound Waterfront (*n* = 15, 8 August 2014), (4) Lonesome Cove (*n* = 17, 10 August 2014), (5) Pile Point (*n* = 44, 12 August 2014), (6) Point Caution (*n* = 48, 7 August 2014), (7) Reuben Tarte (*n* = 14, 10 August 2014), (8) Richardson (*n* = 6, 11 August 2014), (9) Rosario (*n* = 104, 12 July 2014), 10) Strathmann's Beach (*n* = 23, 7 August 2014), (11) Yellow Island (*n* = 56, 12 July 2014), 12) Ruston Way (*n* = 12, 15 July 2014), (13) Hyde (*n* = 3, 14 July 2015), (14) Point Defiance (*n* = 43, 30 January 2014), (15) Titlow Beach (*n* = 47, 1 January 2014) and (16) Starfish Point (*n* = 143, 21 January 2015).

Fixed plots were surveyed throughout the entire epizootic in the intertidal zone at 16 sites within the San Juan Islands (SJI), South Puget Sound (SPS) and the outer coast (OC) of WA between 31 December 2013 and 17 July 2015 (figure 2*a–c* and electronic supplementary material, table S1). Sites were chosen where stars were present, and plots were placed within these sites to represent typical habitats and densities of the population. Plots were established in the majority of the sites and varied in size, owing to the availability of suitable habitat (see electronic supplementary material). During each survey, all ochre stars within the fixed plots were counted, measured and assessed for disease severity [11]. Ochre stars were classified as diseased if they had lesions or loss of turgor owing to tissue breakdown. Continuous water temperature (every 30 min) was collected from temperature loggers (HOBO^®^, Bourne, MA) placed at 0 m mean lower low water at five of the sites. The survey sites represent a range of intertidal ochre star habitats found in the San Juan Islands and South Sound, WA [26]. Ochre stars under 75 mm radius were considered juveniles as individuals below this size are not reproductive. While some ochre stars above this size may not be reproductive, this was the best conservative estimate available for reproductive status without lethally sampling individuals [27].

We used logistic time-series regression to evaluate the effects of ochre star size and temperature on the probability of being diseased in SJI and SPS. Sampling was not frequent enough in OC to analyse these data with a time-series regression. For SJI and SPS, we used a Fourier term with one harmonic (e.g. one sine and one cosine curve) to model the cyclical patterns of season over time (R package ‘tsModel’). The seasonal patterns were different enough between the two geographical regions that model convergence was not possible with both regions in the same model. Therefore, we fit these regions in separate models. Once we fitted harmonic functions to these models, we then added terms for sea star size and included site as a random intercept. For the SPS model, we had to limit the data to surveys conducted in 2013 and 2014, as a nine month time gap between surveys in 2014 and 2015 prevented us from successfully modelling the time effect. We were able to use all of the data in the SJI model. Models were fit with sea star size data and with site as a random intercept. The model was run in R (v. 3.1.2. package ‘lme4’). Marginal *R*^2^- and conditional *R*^2^-values were calculated for each model using the ‘piecewiseSEM’ package [28]. The marginal *R*^2^ describes the proportion of variance explained by the fixed factors alone, whereas the conditional *R*^2^ describes the variance explained by fixed and random factors.

Owing to the placement of loggers, temperature data were only available for five sites in SJI from June to December 2014. We ran a time-series regression on this subset of data in order to understand the effect of temperature on the probability of being diseased. For this time period, which included four sampling events, at each of the sites, we tried fitting the time component of the model in two ways; we used a single harmonic function as described in the logistic regressions above. We also tried fitting the time component with linear and quadratic terms for the centred time data. These models yielded nearly identical coefficients for temperature and size effects, and so we report the results of the latter model, which is more intuitive. Because the sites were intertidal, temperatures could be separated into submerged and exposed temperatures. We tried fitting several terms for temperature: the mean submerged temperature, mean exposed temperature, maximum submerged temperature and maximum exposed temperature for each site and time. Each of these values was calculated from the 5 days preceding the sampling event of interest. We then used Akaike information criteria (AIC) to select the best-fitting model. Site was included as a random effect within the model and temperature was nested within site.

The second set of analyses of the field data were conducted to understand how the demographics of the study populations changed over time. Using the 16 surveyed populations, we used paired Welch two sample *t*-tests (‘*t*.test’ function in the ‘stats' package in R) to compare first, the population size and, second, the mean body radius of sea stars surveyed prior to the outbreak in 2014 and at least 1 year later, after the outbreak. To further understand changes in population size, we compared changes in the number of small (less than 75 mm) and large (greater than 75 mm) sea stars across all of the sites. The timing of the outbreak differed between the regions surveyed. Therefore, comparisons were made for sites: in South Puget Sound between January 2014 and May 2015, in the San Juan Islands between June 2014 and July 2015 and on the OC between June 2014 and June 2015. R code and datasets are available as electronic supplementary material for all field survey analyses.

### (b) Experiment

A controlled experiment was conducted at University of Washington's Friday Harbor Laboratories (Friday Harbor, WA) to understand how temperature and life stage (juveniles and adults) influenced health and survival of sea stars that were previously exposed to SSWD. Thirty-six asymptomatic adult ochre stars (116 ± 14 mm radius, mean ± s.d.) and 48 juveniles (less than 75 mm radius) were collected on 18 June 2014 at Rosario on Orcas Island at the early stage of the regional epidemic (48.64418252 N, −122.8727728 W, figure 2*b*). Although only visually healthy ochre stars were collected, approximately 10% of the ochre stars at the site had visible signs of wasting disease at the time of collection. Thus, all ochre stars may have been exposed to the pathogen and similar recent environmental conditions. Owing to the large spatial extent of this outbreak extending from southern California into British Columbia, we were unable to procure pathogen naive sea stars.

Collected individuals were placed immediately in 38 l flow-through seawater tanks (three adult and four juvenile ochre stars per tank) and acclimated under ambient conditions (approx. 13.5°C) for 3 days. Juveniles were placed in smaller containers within the same tanks to protect them from predation by adult ochre stars. Water temperatures were adjusted (from ambient) using water baths with aquaria heaters or chillers to create four treatments (12, 14, 16 and 19°C), which represent the range of water temperatures recorded by temperature loggers at the intertidal sites surveyed during peak SSWD prevalence (10.6–23.0°C). Each temperature treatment was replicated three times for a total of 12 tanks. Tanks received a constant, controlled flow of unfiltered seawater with a temperature maintained within ± 1°C (s.d.). This system was indoors with controlled air temperature. Separate flow lines were used, so there was no recirculation of water between aquaria. Sea stars were fed frozen mussels once per day. Mussels had been collected from a site without sea stars present. The sea stars were examined daily for signs of wasting disease and death. Individuals were considered dead when arm loss and/or lesions led to loss of turgor and were subsequently removed from experimental tanks. The experiment was terminated after 19 days.

We used a mixed-effects Cox's proportional hazards model (package ‘coxme’ in R v. 3.1.2) to analyse the effect of temperature on (i) the time to disease signs and (ii) the time between disease signs and mortality. In these analyses, tank was included as a random effect. Because temperature did not appear to have a linear effect on either outcome (disease signs or death), we included the temperature treatments as factors as opposed to a continuous numeric variable. We ran the models separately for juveniles and adults as otherwise the assumption of proportionality was not met. This assumption was tested by graphing the Schoenfield residuals of the model (run without random effects) against time (using the ‘cox.zph’ function in the ‘survival’ package in R). Analysis of variance revealed that there were no significant differences between the models with and without the random effect (both *p* > 0.1), so this was a valid approach. R code and datasets are available as electronic supplementary material for these analyses.

## 3. Results

### (a) Field surveys

**Figure 3.**
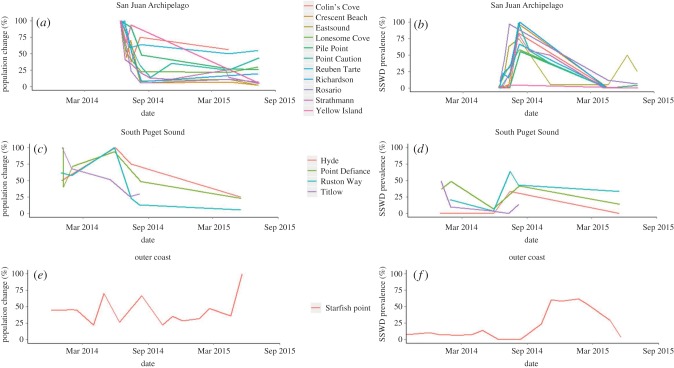
Proportional population decline in (*a*) San Juan Islands (SJI), (*b*) South Puget Sound (SPS), (*c*) outer coast (OC) and disease prevalence (*d*) SJI, (*e*) SPS and (*f*) OC of *P. ochraceus*. SJI and SPS graphs are for adult (>75 mm) ochre stars; OC graphs show data including both adults and juveniles, due to data constraints. Proportional population is scaled to the maximum number of ochre stars observed over the study period.

A total of 6568 ochre stars were surveyed across the 16 study sites between 31 December 2013 and 17 July 2015. All study regions experienced short, severe epizootics, though the timing and magnitude of peak SSWD prevalence varied (figure 3*a–f*). The SJI region, where no epizootic had been documented prior to the study period, had the most severe outbreak of the regions, with 74.0 ± 6.8% (mean ± s.e.) of sea stars showing disease signs during the peak in August 2014. Starfish Point also experienced high SSWD prevalence (61.5 ± 4.1%), peaking in January 2015. Sites within the SPS region had the lowest peak prevalence of the regions studied (14.9 ± 7.5%) in August 2014 after an earlier epizootic in the winter of that year; peak prevalence was 45% at two sites (Titlow and Point Defiance) and 70% at one site (Ruston Way). Only a single diseased ochre star was observed during a year and a half of surveys at Hyde.

The models for the longitudinal time-series data included 5388 observations from SJI and 957 from SPS. Size increased the probability of disease in both regions (table 1). A 10 mm increase in size caused an increase in the odds of disease by 1.23, and 1.15 at SJI and SPS, respectively. The significant effects of the harmonic terms indicate that temporal correlations above and beyond those associated with temperature also contributed to the probability of disease. The conditional and marginal *R*^2^ for the SJI model were 0.691 and 0.54, respectively. The conditional and marginal *R*^2^ for the SPS model were 0.31 and 0.22, respectively. The relatively small differences between the conditional and marginal *R*^2^ values in these models indicates that size and time explained more of the variation in disease status than the random site effects.

**Table 1.** Results from logistic regression of disease status in ochre stars in the San Juan Islands and South Puget Sound between December 2013 and August 2015. In both cases, site was included as a random intercept. Harmonics (sine and cosine curves) were included in both time-series datasets to account for time-dependent effects.

	estimate	s.e.	*z*-value	*p*-value
San Juan Islands				
intercept	−6.77	0.47	−14.5	<0.0000001
sea star radius (mm)	0.021	0.001	14.2	<0.0000001
harmonic 1	−4.96	0.20	−24.6	<0.0000001
harmonic 2	−2.33	0.17	−13.6	<0.0000001
South Puget Sound				
intercept	−2.37	0.36	−6.61	<0.0000001
sea star radius (mm)	0.014	0.002	6.03	<0.0000001
harmonic 1	−0.33	0.23	−1.43	0.15
harmonic 2	0.43	0.13	3.41	0.0007

**Figure 4.**
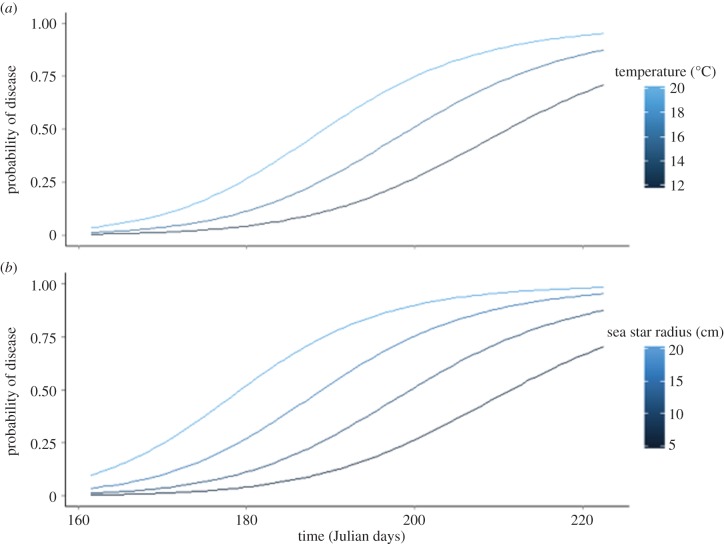
Predicted probability of SSWD in SJI from June through August 2014 as a function of temperature (*a*) and size (*b*). Predictions are based on the SJI temperature model (table 2). Temperature predictions are for ochre stars with 10 cm radius, and size predictions are for 16°C.

The subset of SJI data where temperature data were also available contained 2012 data points from five sites sampled between June and August 2014. The best-fitting model from this dataset included linear and quadratic terms for day and day squared as well as terms for ochre star size and mean submerged temperature (table 2 and electronic supplementary material). For every 1°C increase in temperature, the probability of disease increased by 1.30 (mean; figure 4*a*). For every 10 mm increase in size, the probability of disease increased by 1.23 (figure 4*b*). The marginal *R*^2^ for this model was 0.50, and the conditional *R*^2^ was 0.59.

**Table 2.** Results from the logistic regression of the time-series data for the five SJI sites for which temperature data was available. Terms, coefficients and *p*-values from the best-fitting model are in (*a*). Site was included as a fixed effect in this model and Crescent Bay was used as the baseline site from which all comparisons were made. Akaike information criteria (AIC) for all temperature models tested (*b*). Temperature was included as a random effect nested within the random effect of site for all models tested. Submerged temperatures are when sites were below the seawater level and exposed are when sites were above the seawater level. All temperature data were either maximums or means of these values from the 5 days previous to the sea star survey.

(*a*)—term	estimate	s.e.	*z*-value	*p*-value
intercept	−8.68	1.03	−8.45	<0.0000001
sea star radius (cm)	0.214	0.030	7.01	<0.0000001
sea temp. (°C, 5-day avg.)	0.261	0.055	4.76	<0.00001
day (centred)	0.118	0.025	4.658	<0.00001
day (centred)^2^	−4.941	6.783	−0.728	0.446

(*b*)—model				AIC
day + day^2^ + size + mean subm. temp				1275.7
day + day^2^ + size + max. subm. temp				1286.0
day + day^2^ + size				1287.4
day + day^2^ + size + max. exposed temp				1288.1
day + day^2^ + size + mean. exposed temp				1288.9

**Figure 5.**
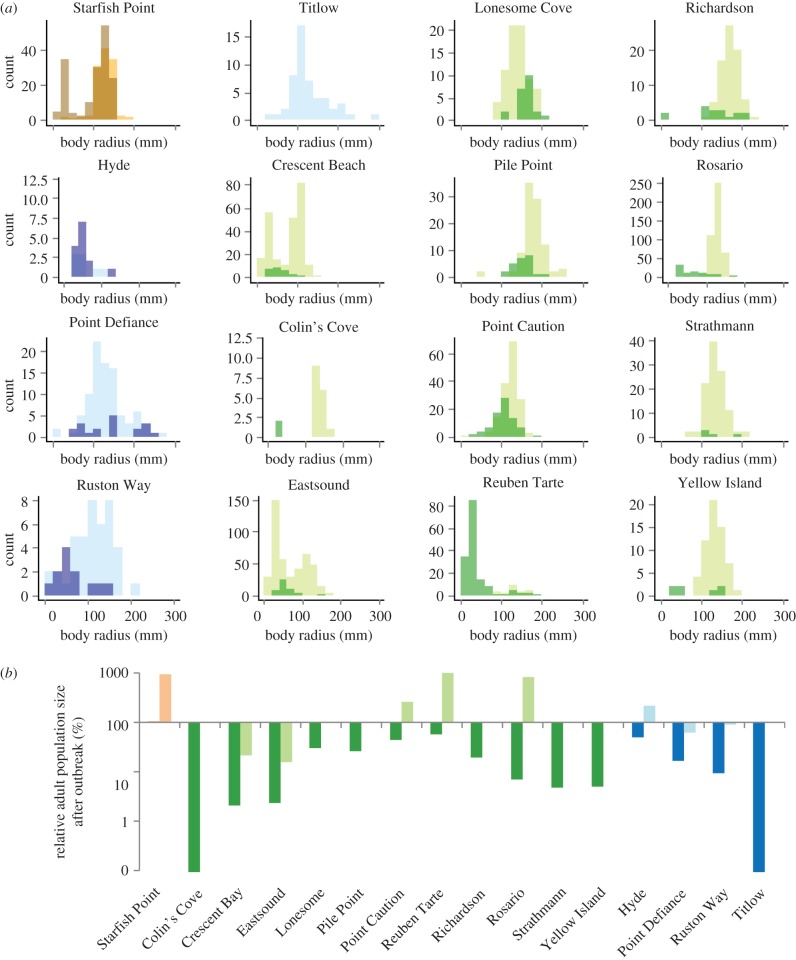
Demography of ochre stars before and after the SSWD epidemic. (*a*) Light shades represent 2014 data (before outbreak) and dark shades represent 2015 data (after outbreak). Colours represent geographical regions surveyed. Blue, South Puget Sound (January 2014 and May 2015); green, San Juan Islands (June 2014 and July 2015); orange, outer coast (June 2014 and June 2015). (*b*) Populations of adults (dark) and juveniles (light) relative to the pre-outbreak populations.

Ochre star populations declined by 67% on average over our study period (*t*_19.3_ = 2.34, *p* = 0.02; figures 3 and 5*a*). This was driven by 80% reductions in adult populations (*t*_17.6_ = 3.03, *p* = 0.008; figure 5*b*). Juvenile populations did not change significantly in population size (*t*_21.5_ = 3.03, *p* = 0.86). The impact of the outbreak on population size varied by site, and adult ochre stars had an abundance of zero at Colin's Cove (SJI) and Titlow Beach (SPS) after the outbreak. In contrast to the SJI and SPS regions, the OC site experienced a 38% increase in ochre star abundance, which consisted of increases in both juvenile and adult stars. The average radius of ochre stars decreased from 119 to 86 mm after the outbreak (*t*_25.85_ = 2.287, *p* = 0.031).

### (b) Experiment

**Figure 6.**
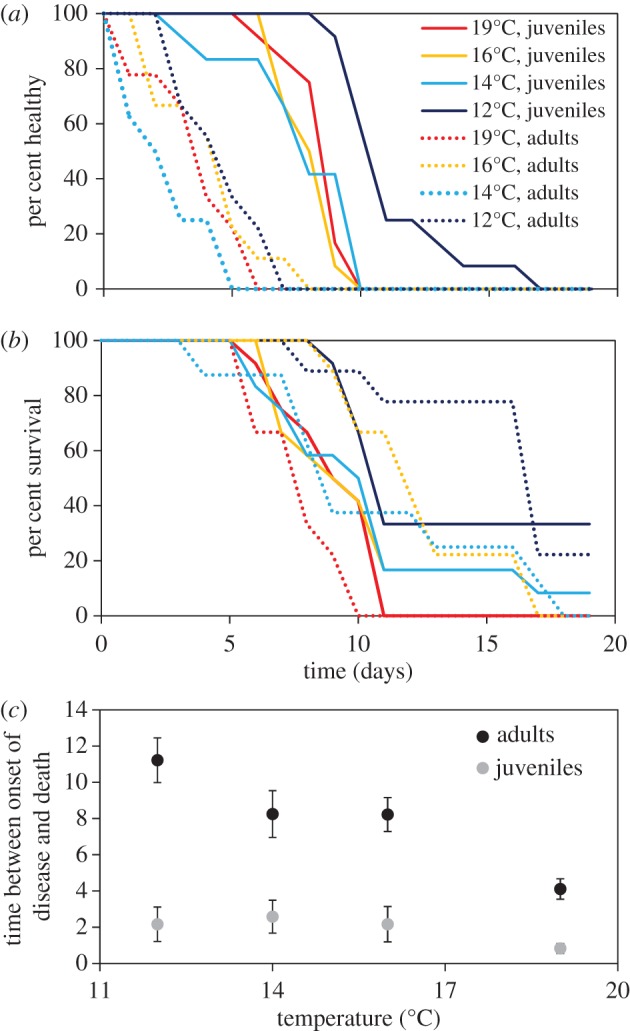
*Pisaster ochraceus* at four different temperatures in a controlled experiment. Differential rates of disease progression (*a*), survival (*b*) and time between onset of disease signs and death (*c*) compared in adult (>75 mm radius) and juvenile (<75 mm radius). Time to disease onset and survivorship are affected by reproductive stages and temperature.

All juvenile and adult ochre stars developed signs of SSWD and died of disease during the 19-day experiment (table 3 and figure 6*a–c*). The development of disease signs was significantly affected by temperature for juveniles, but not adults. For juvenile individuals, the hazard of developing disease signs increased by 7% at 14**°**C, 11.8% at 16**°**C and 9.7% at 19**°**C relative to the hazard of developing disease signs at 12**°**C. While we were not able to test it statistically, juveniles showed signs of disease earlier than adults. It took 5–8 days before all juveniles in a temperature treatment showed disease signs, whereas it took 10–12 days for all adults in a temperature treatment to show disease signs.

**Table 3.** Results of survival analyses from temperature experiment that showed significant effects. This includes the effect of temperature on the hazard of juvenile ochre stars developing disease signs and the effect of temperature on the hazard of diseased adult ochre stars dying. In both models, temperature effects are relative to the coldest temperature treatment, 12°C.

	estimate	exponent (estimate)	s.e. (exponent (estimate))	*z*-value	*p*-value
*hazard of juvenile ochre stars developing disease signs*					
* 14°C*	*1.94*	*6.95*	*0.55*	*3.53*	*<0.001*
*16°C*	*2.47*	*11.8*	*0.58*	*4.28*	*<0.0001*
*19°C*	*2.27*	*9.73*	*0.58*	*3.93*	*<0.0001*
*hazard of death for adult ochre stars with disease signs*					
14°C	1.10	3.01	0.68	1.62	0.1
16°C	1.30	3.66	0.69	1.91	0.056
*19°C*	*2.90*	*18.23*	*0.75*	*3.85*	*<0.0005*

The time period between development of disease signs and death was influenced by temperature for adults, but not juveniles. The risk of death in diseased stars at 14 and 16**°**C was not significantly different from the risk of death at 12**°**C. However, the risk of death at 19**°**C was 18% greater relative to the risk of death at 12**°**C. While larger ochre stars showed disease signs sooner than juveniles, disease juveniles died more quickly than diseased adults.

## 4. Discussion

This study focuses on the variation in timing and scale of the SSWD epizootic in three regions of Washington state and documents large impacts to populations of the dominant intertidal species, *P. ochraceus*. SSWD was first reported on the OC of Washington in 2013; however, the peak prevalence did not occur there until winter 2015. In contrast, there were two distinct outbreaks of the disease in the south Puget Sound, first in early winter 2014 and later in mid-summer 2014. The epidemic in the San Juan Islands started a full year after the first reports and was more severe, decimating most populations monitored in the summer of 2014. In the San Juan Islands, adult ochre star abundances declined to only one quarter of their pre-outbreak abundance.

In field populations, larger individuals had both greater probabilities of being observed with disease and greater reductions in abundance after the outbreak. This pattern is partially reflected in the laboratory experiment, where adults developed signs of disease more quickly. However, once the juveniles developed signs of infection, they died in two days. Therefore, the lower probability of disease in juvenile ochre stars in the field likely reflects a lower initial disease risk as well as recruitment and a lower likelihood of observing diseased individuals because they die more quickly. The net result of these size effects was a drop in reproductive adults to one quarter of their pre-outbreak populations and a shift towards a smaller size structure in the population.

The dramatic reductions of larger adult sea stars in this keystone species may have lasting impacts on population recovery and the composition of rocky intertidal communities. Pre-epidemic studies on ochre stars in the Salish Sea showed that both the average size of populations and size structure within populations were stable during the previous 43 years [26,27,29]. This steady baseline contrasts sharply with the population shifts found during the epidemic. The changes in juvenile ochre stars varied considerably among sites. Five sites showed increases in juvenile ochre star populations, whereas four showed decreases. This suggests that complex meta-population structure may affect recruitment among sites and provides hope that some sites may be already recovering from declines of adult ochre stars; however, previous studies show that survival of ochre stars in the year following recruitment can be highly variable and may be critical for survival to adulthood in this species [30]. Future monitoring should prioritize identification of source populations of ochre stars that should be conservation priorities and may reveal the extent to which the recruitment of juvenile sea stars seen at some sites contributes to recovery.

Consistent with some other marine diseases, results from this study suggest that changes in ocean temperature might hasten outbreak progression and the overall severity of impacts. However, in the ocean, increased and more variable temperatures have been associated with disease outbreaks in numerous species, including corals [31–34], sea urchins [35], abalones and other shellfish [36], finfish [37] and marine mammals [14,38]. Water temperature was identified as a facilitator in previous SSWD outbreaks [16,17,39]. In our field surveys, warmer temperatures were associated with a greater probability of being observed with disease. This suggests that sites such as Rosario, in East Sound, which, owing to low levels of mixing can reach summer sea surface temperatures of 23**°**C, might be especially susceptible to SSWD. This geographical susceptibility, compounded with the regionally high temperatures in the summer of 2014, which was the warmest on record in the northeast Pacific, could have exacerbated the outbreak [40]. In particular, the accumulated June–August 2014 anomaly shows sea surface temperature was very warm in the SJI, where the disease outbreak was most severe, but not on the OC of Washington or lower Puget Sound. The faster progression of disease in ochre stars in the laboratory experiment also supports the hypothesis that anomalously warm temperatures in the SJI contributed to the higher rates of disease relative to the OC and lower Puget Sound. Temperature increased both the hazard of presenting disease signs for juveniles and the hazard of death for diseased adults. Regardless, increased mortality rate has opposing effects on disease dynamics, increasing the impact of disease during an epidemic, but potentially causing a disease to burn out owing to the depletion of infected individuals in the population.

In addition to anomalously warm sea surface temperatures, June, July and August spring tides in the San Juan Islands and lower Puget Sound were low during the daytime, leading to full sun exposure during the warmest parts of the day (i.e. between mid-morning and early afternoon). In contrast, on the OC, low tides occurred in the early morning during the summers, resulting in lower daytime air temperatures when intertidal sea stars were exposed. Interestingly, the air temperature to which SJI sites were exposed was not a significant predictor of disease in our models, suggesting that ochre stars may be avoiding warm air temperatures or that these exposures are too short to have a lasting impact. Further studies comparing regional variation in sea temperature changes and air temperature during exposure may show whether exposure contributes to the regional variation in disease prevalence and timing.

The mechanisms for temperature facilitation of disease progression in this host–pathogen interaction are currently unknown. Most obviously, mortality rates of most organisms (whether infected or not) tend to increase with temperature. Field and experimental studies suggest that ochre stars are susceptible to metabolic stress at high water temperatures [41–43]. Thermoregulation of ochre stars is compromised when temperatures are more than 16°C, owing to lower coelomic fluid volumes [42]. In addition, metabolic demands on ochre stars are greater at warmer temperatures (50% greater at 15°C versus 10°C [44]). Furthermore, warming water temperatures and altered temperature variation above a species' thermal optimum can immunosuppress hosts, thereby changing their susceptibility to or tolerance of infection. Warm temperature could also increase pathogen proliferation up to a point [14,40,45]. SSWD-affected tissues show pronounced tissue degradation and proteasomal activities, suggesting that induced necrosis of animals may result from SSaDV exposure [46]. Increased expression of immune and tissue repair genes after infection with SSWD indicates sea stars mount a robust, and likely energetically costly, immune response to SSaDV [46,47]. Further studies examining how thermal stress, disease signs and energetically costly immune responses interact may reveal the mechanisms underlying temperature dependence in SSWD.

The rapidity and unexpected nature of this outbreak leaves us with many knowledge gaps to fill about mechanisms underlying the timing and severity of the outbreak, the role of seasonal and environmental shifts in facilitating disease and the long-term impacts of SSWD on ochre star populations and intertidal communities. While our models were able to explain some of the factors that predict disease status of sea stars, the *R*^2^ values for our models indicate that other, unknown factors are also contributing to disease. Potential factors include hydrodynamic conditions, multi-host community structure, host density and pathogen strain. One of the bigger mysteries is in understanding the changes that precipitated the shift to a wide host-range and increased the prevalence of this disease, because an outbreak of SSWD on this scale has not previously been observed despite records of the virus from museum specimens. It is unknown yet whether the shifts could be due to new, more virulent pathogen strains, changing environmental conditions, altered structure of host populations or some combination. Continuing studies in the within- and between-host dynamics of this disease are critical for understanding how diseases with previously low-impact local effects can become high-impact multi-host pandemics.

One of the concerns about this outbreak is in the impact it will have on community dynamics. Ochre stars prey primarily on mussels, but are also known to opportunistically feed on various gastropods, barnacles and even chitons [24,48]. A possible impact of this population decline is a large influx of mussels, with resultant changes in the community as mussels overgrow other primary space holders, as has been repeatedly shown with experimental removal of ochre stars [24,25,49]. These predictions are complicated by declines of several other keystone predators in this epizootic. For example, SSWD-induced declines of the sunflower star, *Pycnopodia helianthoides*, and the mottled star, *Evasterias troschelii*, will also contribute to release of prey.

In the ocean, there are relatively few reports of large-scale epidemics of multi-host pathogens (but see [5,50]). Similar outbreaks in impact and host range to the current SSWD event have been observed in frogs affected by chytridiomycosis, crustaceans impacted by white spot syndrome and bats affected by white-nose syndrome. The rate of mortality at some of our sites compares with that in some specific outbreaks in the amphibian chytridiomycosis pandemic. For example, during a California outbreak in the yellow-legged frog species complex (*Rana muscosa* and *R. serrae*), 100% of the frogs from the species complex died in just over a year [51]. In central Panama alone, approximately 30 species were completely eradicated owing to the disease [52]. While there are large impacts to the ochre star populations, there are even greater impacts to the most susceptible sea star species, *Pycnopodia helianthoides*, which may be at risk of endangerment (M. E. Eisenlord, C. D. Harvell, M. Turner 2014, personal observation).

In conclusion, longitudinal monitoring revealed a steeply accelerating epidemic of SSWD and mass mortality in Washington State that was more pronounced in the San Juan Islands and more attenuated on the OC and lower Puget Sound. Warm temperatures were associated with prevalence in the field and increased the rate of developing disease signs and dying in laboratory experiments, leading us to suggest the anomalously warm temperatures of 2014 increased the impact of this epizootic on the sea star population. The population impact of this epidemic could cascade for years, even if the epizootic ends before killing the remaining survivors. Despite their low abundances, the presence of some asymptomatic ochre stars in 2015 at the majority of the survey sites and the recruitment of juveniles to some of the sites leaves us hopeful for recovery in the region. The rapid population declines associated with SSWD follows a classic manifestation of a highly virulent, multi-host pathogen. Given the fast progression of the outbreak and the dramatic population impacts it can have, understanding the factors that drive SSWD continues to be a research priority.

## Supplementary Material

Description of supplementary documents.pdf

## Supplementary Material

Site data & Temp anomaly.pdf

## Supplementary Material

Field Survey Logistic Regression.R

## Supplementary Material

SSWD demographic analysis-2.R

## Supplementary Material

SSWD Experiment.R

## Supplementary Material

Demography_SPS_SJI_SP-2.txt

## Supplementary Material

SSWD All Pisaster.txt

## Supplementary Material

SSWD_SJI_Temp.txt

## Supplementary Material

Pisaster_Temp_Exp_R.txt

## Data accessibility

The datasets supporting this article have been provided as part of the electronic supplementary material.

## References

[1] Parrish CR, Holmes EC, Morens DM, Park E-C, Burke DS, Calisher CH, Laughlin CA, Saif LJ, Daszak P 2008 Cross-species virus transmission and the emergence of new epidemic diseases. Microbiol. Mol. Biol. Rev. 72, 457–470. (10.1128/MMBR.00004-08) 18772285 PMC2546865

[2] Woolhouse ME, Haydon DT, Antia R 2005 Emerging pathogens: the epidemiology and evolution of species jumps. Trends Ecol. Evol. 20, 238–244. (10.1016/j.tree.2005.02.009) 16701375 PMC7119200

[3] Woolhouse MEJ, Gowtage-Sequeria S 2006 Host range and emerging and reemerging pathogens. In *Ending the war metaphor: the changing agenda for unraveling the host-microbe relationship – workshop summary*, pp. 192–206. Washington, DC: National Academies Press.

[4] Fisher MC, Garner TW, Walker SF 2009 Global emergence of *Batrachochytrium dendrobatidis* and amphibian chytridiomycosis in space, time, and host. Annu. Rev. Microbiol. 63, 291–310. (10.1146/annurev.micro.091208.073435) 19575560

[5] Escobedo-Bonilla CM, Alday-Sanz V, Wille M, Sorgeloos P, Pensaert M, Nauwynck H 2008 A review on the morphology, molecular characterization, morphogenesis and pathogenesis of white spot syndrome virus. J. Fish. Dis. 31, 1–18. (10.1111/j.1365-2761.2007.00877.x) 18086030

[6] Lafferty KD, Harvell CD, Conrad JM, Friedman CS, Kent ML, Kuris AM, Powell EN, Rondeau D, Saksida SM 2015 Infectious diseases affect marine fisheries and aquaculture economics. Annu. Rev. Mar. Sci. 7, 471–496. (10.1146/annurev-marine-010814-015646) 25251276

[7] Jones KE, Patel NG, Levy MA, Storeygard A, Balk D, Gittleman JL, Daszak P 2008 Global trends in emerging infectious diseases. Nature 451, 990–993. (10.1038/nature06536) 18288193 PMC5960580

[8] Daszak P, Cunningham AA, Hyatt AD 2003 Infectious disease and amphibian population declines. Divers. Distrib. 9, 141–150. (10.1046/j.1472-4642.2003.00016.x) PMC2640803 10603206

[9] Anderson R, Gordon D 1982 Processes influencing the distribution of parasite numbers within host populations with special emphasis on parasite-induced host mortalities. Parasitology 85, 373–398. (10.1017/S0031182000055347) 7145478

[10] Raffel TR, LeGros RP, Love BC, Rohr JR, Hudson PJ 2009 Parasite age-intensity relationships in red-spotted newts: does immune memory influence salamander disease dynamics? Int. J. Parasitol. 39, 231–241. (10.1016/j.ijpara.2008.06.011) 18708064

[11] Raffel TR, Lloyd-Smith JO, Sessions SK, Hudson PJ, Rohr JR 2011 Does the early frog catch the worm? Disentangling potential drivers of a parasite age–intensity relationship in tadpoles. Oecologia 165, 1031–1042. (10.1007/s00442-010-1776-0) 20852894 PMC3057004

[12] Groner ML, Rollins-Smith LA, Reinert LK, Hempel J, Bier ME, Relyea RA 2014 Interactive effects of competition and predator cues on immune responses of leopard frogs at metamorphosis. J. Exp. Biol. 217, 351–358. (10.1242/jeb.091611) 24115058

[13] Roberts CM, Hawkins JP 1999 Extinction risk in the sea. Trends Ecol. Evol. 14, 241–246. (10.1016/S0169-5347(98)01584-5) 10354629

[14] Burge CA et al. 2014 Climate change influences on marine infectious diseases: implications for management and society. Annu. Rev. Mar. Sci. 6, 249–277. (10.1146/annurev-marine-010213-135029) 23808894

[15] Maynard J et al. 2016 Improving marine disease surveillance through sea temperature monitoring, outlooks and projections. Phil. Trans. R. Soc. B 371, 20150208 (10.1098/rstb.2015.0208) 26880840 PMC4760138

[16] Bates AE, Hilton BJ, Harley CD 2009 Effects of temperature, season and locality on wasting disease in the keystone predatory sea star *Pisaster ochraceus*. Dis. Aquat. Organ. 86, 245–251. (10.3354/dao02125) 20066959

[17] Staehli A, Schaerer R, Hoelzle K, Ribi G 2009 Temperature induced disease in the starfish *Astropecten jonstoni*. Mar. Biodivers. Rec. 2, e78 (10.1017/S1755267209000633)

[18] Eckert GL, Engle JM, Kushner DJ. 2000 Sea star disease and population declines at the Channel Islands. In *Proc. 5th California Islands symposium, Santa Barbara, CA, 29 March–1 April 1999*. Washington, DC: US Minerals Management Service.

[19] Bond NA, Cronin MF, Freeland H, Mantua N 2015 Causes and Impacts of the 2014 Warm anomaly in the NE Pacific. Geophys. Res. Lett. 42, 3414–3420. (10.1002/2015GL063306)

[20] Hartmann DL 2015 Pacific sea surface temperature and the winter of 2014. Geophys. Res. Lett. 42, 1894–1902. (10.1002/2015GL063083)

[21] MARINe. 2015 Sea Star Wasting Syndrome 2015 [03/15/2015]. See http://www.eeb.ucsc.edu/pacificrockyintertidal/data-products/sea-star-wasting/index.html.

[22] Hewson I et al. 2014 Densovirus associated with sea-star wasting disease and mass mortality. Proc. Natl Acad. Sci. USA 111, 17 278–17 283. (10.1073/pnas.1416625111) PMC4260605 25404293

[23] Menge BA 1979 Coexistence between the seastars *Asterias vulgaris* and *A*. *forbesi* in a heterogeneous environment: a non-equilibrium explanation. Oecologia 41, 245–272. (10.1007/BF00377430) 28309763

[24] Paine RT 1966 Food web complexity and species diversity. Am. Nat. 100, 65–75. (10.1086/282400)

[25] Menge BA, Berlow EL, Blanchette CA, Navarrete SA, Yamada SB 1994 The keystone species concept: variation in interaction strength in a rocky intertidal habitat. Ecol. Monogr. 64, 249–286. (10.2307/2937163)

[26] Rogers TL, Elliott JK 2013 Differences in relative abundance and size structure of the sea stars *Pisaster ochraceus* and *Evasterias troschelii* among habitat types in Puget Sound, Washington, USA. Mar. Biol. 160, 853–865. (10.1007/s00227-012-2139-7)

[27] Menge BA 1974 Effect of wave action and competition on brooding and reproductive effort in the seastar, *Leptasterias hexactis*. Ecology 55, 84–93. (10.2307/1934620)

[28] Lefcheck JJ In press. Piecewise SEM: piecewise structural equation modeling in R for ecology, evolution, and systematics. Methods Ecol. Evol. (10.1111/2041-210X.12512)

[29] Menge BA 1972 Competition for food between two intertidal starfish species and its effect on body size and feeding. Ecology 55, 635–644. (10.2307/1934777)

[30] Sewell M, Watson J 1993 A ‘source’ for asteroid larvae? Recruitment *of Pisaster ochraceus*, *Pycnopodia helianthoides* and *Dermasterias imbricata* in Nootka Sound, British Columbia. Mar. Biol. 117, 387–398.

[31] Bruno JF, Selig ER, Casey KS, Page CA, Willis BL, Harvell CD, Sweatman H, Melendy AM, Roberts C 2007 Thermal stress and coral cover as drivers of coral disease outbreaks. PLoS Biol. 5, e124 (10.1371/journal.pbio.0050124) 17488183 PMC1865563

[32] Ward JR, Kim K, Harvell C 2007 Temperature affects coral disease resistance and pathogen growth. Mar. Ecol. Prog. Ser. 329, 115–121. (10.3354/meps329115)

[33] Boyett HV, Bourne DG, Willis BL 2007 Elevated temperature and light enhance progression and spread of black band disease on staghorn corals of the Great Barrier Reef*.* Mar. Biol. 151, 1711–1720. (10.1007/s00227-006-0603-y)

[34] Dalton S, Godwin S, Smith S, Pereg L 2010 Australian subtropical white syndrome: a transmissible, temperature-dependent coral disease. Mar. Freshw. Res. 61, 342–350. (10.1071/MF09060)

[35] Feehan CJ, Scheibling RE 2014 Effects of sea urchin disease on coastal marine ecosystems. Mar. Biol. 161, 1467–1485. (10.1007/s00227-014-2452-4)

[36] Ben-Horin T, Lenihan HS, Lafferty KD 2013 Variable intertidal temperature explains why disease endangers black abalone. Ecology 94, 161–168. (10.1890/11-2257.1) 23600250

[37] Bowden TJ 2008 Modulation of the immune system of fish by their environment. Fish Shellfish Immunol. 25, 373–383. (10.1016/j.fsi.2008.03.017) 18562213

[38] Burek KA, Gulland FM, O'Hara TM 2008 Effects of climate change on Arctic marine mammal health. Ecol. Appl. 18, S126–SS34. (10.1890/06-0553.1) 18494366

[39] Dungan ML, Miller TE, Thomson DA 1982 Catastrophic decline of a top carnivore in the Gulf of California rocky intertidal zone. Science 216, 989–991. (10.1126/science.216.4549.989) 17809070

[40] Ocean Networks Canada. *Northeast Pacific warming 2014* (accessed 9 September 2015). See http://www.oceannetworks.ca/northeast-pacific-warming.

[41] Petes LE, Mouchka ME, Milston-Clements RH, Momoda TS, Menge BA 2008 Effects of environmental stress on intertidal mussels and their sea star predators. Oecologia 156, 671–680. (10.1007/s00442-008-1018-x) 18347815

[42] Pincebourde S, Sanford E, Helmuth B 2009 An intertidal sea star adjusts thermal inertia to avoid extreme body temperatures. Am. Nat. 174, 890–897. (10.1086/648065) 19827942

[43] Monaco CJ, Wethey DS, Helmuth B 2014 A dynamic energy budget (DEB) model for the keystone predator *Pisaster ochraceus*. PLoS ONE 9, e104658 (10.1371/journal.pone.0104658) 25166351 PMC4148243

[44] Fly EK, Monaco CJ, Pincebourde S, Tullis A 2012 The influence of intertidal location and temperature on the metabolic cost of emersion in *Pisaster ochraceus*. J. Exp. Mar. Biol. Ecol. 422, 20–28. (10.1016/j.jembe.2012.04.007)

[45] Harvell CD et al. 2002 Climate warming and disease risks for terrestrial and marine biota. Science 296, 2158–2162. (10.1126/science.1063699) 12077394

[46] Gudenkauf B, Hewson I 2014 Metatranscriptomic Analysis of *Pycnopodia helianthoides* (Asteroidea) affected by sea star wasting disease. PLoS ONE 10, e0128150 (10.1371/journal.pone.0128150) PMC4447261 26020776

[47] Fuess LE et al. 2015 Up in arms: immune and nervous system response to sea star wasting disease. PLoS ONE 10, e0133053 (10.1371/journal.pone.0133053) 26176852 PMC4503460

[48] Harley C, Pankey M, Wares J, Grosberg R, Wonham M 2006 Color polymorphism and genetic structure in the sea star *Pisaster ochraceus*. Biol. Bull. 211, 248–262. (10.2307/4134547) 17179384

[49] Paine RT 2002 Trophic control of production in a rocky intertidal community. Science 296, 736–739. (10.1126/science.1069811) 11976455

[50] Burge CA, Friedman CS 2012 Quantifying ostreid herpesvirus (OsHV-1) genome copies and expression during transmission. Microb. Ecol. 63, 596–604. (10.1007/s00248-011-9937-1) 21935610

[51] Vredenburg VT, Knapp RA, Tunstall TS, Briggs CJ 2010 Dynamics of an emerging disease drive large-scale amphibian population extinctions. Proc. Natl Acad. Sci. USA 107, 9689–9694. (10.1073/pnas.0914111107) 20457913 PMC2906868

[52] Crawford AJ, Lips KR, Bermingham E 2010 Epidemic disease decimates amphibian abundance, species diversity, and evolutionary history in the highlands of central Panama. Proc. Natl Acad. Sci. USA 107, 13 777–13 782. (10.1073/pnas.0914115107) PMC2922291 20643927

