# Supplementary material for: Ochre star mortality during the 2014 wasting disease epizootic: role of population size structure and temperature
Source: Philos Trans R Soc Lond B Biol Sci. 2016 Mar 5;371(1689):20150212. doi: 10.1098/rstb.2015.0212 (PMC4760142; doi:10.1098/rstb.2015.0212)
Supplement: Description of supplementary documents.pdf [file rstb20150212supp1.pdf]

Supplementary material for Eisenlord ME, Groner ML, Yoshioka RM, Elliott J, Maynard J, Fradkin S, Turner M, Pyne K, Rivlin N, van Hooidonk R, Harvell CD. Ochre star mortality during the 2014 wasting disease epizootic: Role of population size structure and temperature, Phil. Trans. R. Soc. B. 10.1098/ rstb.2015.0212

Description of supplementary documents

1_Eisenlord et al 2016_Site data & Temp anomaly.docx: Contains 1) Table S1 with location, coordinates and plot sizes for the 16 survey sites, 2) Sea surface temperature anomaly calculations for figure 2

2_Eisenlord et al 2016_SSWD_Field Survey Logistic Regression.R: R-code for logistic regression of time series data from San Juan Islands (SJI), South Puget Sound (SPS) and the subset of SJI data for which there is temperature data

3_Eisenlord et al 2016_SSWD demographic analysis.R: R-code for Welch's t-test for population size structure before and after outbreak

4_Eisenlord et al 2016_SSWD Experiment.R: R-code for survival analysis in temperature experiment

5_Eisenlord et al 2016_Demography_SPS_SJI_SP.txt: Dataset associated with demographic analysis

6_Eisenlord et al 2016_SSWD All Pisaster.txt: Dataset associated with logistic regression

7_Eisenlord et al 2016_SSWD_SJI_Temp.txt: Dataset associated with logistic regression temperature analysis

8_Eisenlord et al 2016_Pisaster_Temp_Exp_R.txt: Dataset associated with temperature experiment
