# Supplementary material for: Ochre star mortality during the 2014 wasting disease epizootic: role of population size structure and temperature
Source: Philos Trans R Soc Lond B Biol Sci. 2016 Mar 5;371(1689):20150212. doi: 10.1098/rstb.2015.0212 (PMC4760142; doi:10.1098/rstb.2015.0212)
Supplement: Site data & Temp anomaly.pdf [file rstb20150212supp2.pdf]

Supplementary material for Eisenlord ME, Groner ML, Yoshioka RM, Elliott J, Maynard J, Fradkin S, Turner M, Pyne K, Rivlin N, van Hooidonk R, Harvell CD. Ochre star mortality during the 2014 wasting disease epizootic: Role of population size structure and temperature, Phil. Trans. R. Soc. B. 10.1098/ rstb.2015.0212

Contents Page

Table S1. Location and coordinates for the 16 survey sites. 1
Sea surface temperature anomaly calculations for figure 2 2

Table S1. Regional location, site name, island/locality and coordinates and survey area for 16 survey locations in Washington, USA.

Region	#	Site	Locality	Latitude (°N)	Longitude (°W)	Survey area (m^2)
San Juan Islands (SJI)	1	Colin's Cove	San Juan	48.5496003	-123.0058828	297
	2	Crescent Beach	Orcas	48.69110779	-122.8998859	361
	3	Eastsound Waterfront	Orcas	48.69411945	-122.9077676	258
	4	Lonesome Cove	San Juan	48.62106404	-123.1125499	4139
	5	Pile Point	San Juan	48.48236771	-123.0888851	1118
	6	Point Caution	San Juan	48.56198455	-123.0174311	442
	7	Reuben Tarte	San Juan	48.61217486	-123.0981535	1154
	8	Richardson	Lopez	48.44687	-122.89988	621
	9	Rosario	Orcas	48.64418252	-122.8727728	616
	10	Strathmann’s Beach	San Juan	48.56311706	-123.0251518	25
	11	Yellow Island	San Juan Channel	48.59306207	-123.0322087	335
South Puget Sound (SPS)	12	Ruston Way	Tacoma	47.294980822	-122.497940865	300
	13	Hyde	Tacoma	47.27560545	-122.4612332	300
	14	Point Defiance	Tacoma	47.30660093	-122.515690989	1026
	15	Titlow Beach	Tacoma	47.25322675	-122.5529986	837
Outer Washington Coast	16	Starfish Point	Olympic National Park	47.652583	-124.3919835	52

Sea surface temperature anomaly- calculations for figure 2

Methods

The climatology used for the new NOAA Coral Reef Watch (CRW) 5-km Geo-Polar Blended Night-only products was produced using night-only values from 1985-2012 from the NOAA Pathfinder Version 5.2 sea surface temperature (SST) data set (a U.S. official climate data record).12 monthly mean climatologies were produced for the 28-year period and the monthly means were used as the baseline from which we calculated anomalies. The 5-km climatological average values were bias-adjusted to match 5-km SST data available from CRW for the study period. Anomalies were calculated by comparing daily 5-km SST data to the bias-adjusted monthly mean values. The anomaly values shown are the average anomaly calculated for June, July and August, 2014. The anomaly data are useful for viewing large-scale regional patterns in sea temperature anomalies during the study period. However, the data resolution of 5-km is too coarse for use in statistical analyses comparing disease among our intertidal survey sites.

Results

Of the three areas surveyed within the region, average SST anomalies for June-August, 2014 were greatest and in the San Juan Islands. Average anomalies also approached or exceeded 2 °C on the outer WA coast. However, average anomalies >2 °C and even 3 °C were sustained through the summer months of 2014 (June-August) within the San Juan Islands (figure 2).
